# Insulin preserves fast-twitch muscle mass during early STZ-induced diabetes in rats

**DOI:** 10.1016/j.bbrep.2026.102627

**Published:** 2026-05-08

**Authors:** Yuki Yamasaki, Kazuhiro Nomura, Yuka Matsumoto, Kana Fukuda-Takeji, Yumiko Miyatake, Naoya Suemasa, Yuna Izumi-Mishima, Sonoko Yasui-Yamada, Masashi Kuroda, Nagakatsu Harada, Yasuo M. Tsutsumi, Tadahiro Kitamura, Rie Tsutsumi, Yutaka Nakaya, Hiroshi Sakaue

**Affiliations:** a Department of Nutrition and Metabolism, Institute of Biomedical Sciences, Tokushima University Graduate School, Tokushima, Japan; b Fukuoka Women's University, Fukuoka, Japan; c Diabetes Therapeutics and Research Center, University of Tokushima, Tokushima, Japan; d Department of Anesthesiology and Critical Care, Hiroshima University, Hiroshima, Japan; e Metabolic Signal Research Center, Institute for Molecular and Cellular Regulation, Gunma University, Maebashi, Japan; f Department of Health and Nutrition, Baika Women's University, Osaka, Japan

**Keywords:** Skeletal muscle atrophy, Type 1 diabetes mellitus, Insulin, Voluntary exercise

## Abstract

**Purpose:**

Skeletal muscle atrophy is a critical complication of type 1 diabetes mellitus (T1DM), primarily caused by insulin deficiency. While physical activity is known to improve metabolic health, its effectiveness in preventing diabetes-induced muscle atrophy, particularly in the absence of insulin, remains unclear. This study examined the effects of voluntary exercise on muscle atrophy in an early phase streptozotocin (STZ)-induced diabetic rat model.

**Methods:**

STZ was administered to male Wistar rats and SPORTS rats, which maintain high activity levels even after diabetes onset. Blood glucose levels, skeletal muscle mass, and muscle atrophy-related gene expression (Atrogin-1 and MuRF1) were measured, as well as locomotor activity. The effects of insulin, the sodium-glucose cotransporter (SGLT) inhibitor phlorizin, and leucine supplementation were also examined.

**Results:**

Extensor digitorum longus **(**EDL) muscle mass decreased significantly within seven days of diabetes onset, accompanied by early expression of atrophy-related genes. Insulin administration prevented the decrease in EDL muscle mass, whereas phlorizin did not, despite improving hyperglycemia. SPORTS rats maintained high voluntary locomotor activity after diabetes onset but failed to prevent muscle atrophy. Metabolomic analysis indicated altered branched-chain amino acid (BCAA) metabolism in diabetic muscle tissue. Leucine supplementation partially attenuated muscle atrophy in STZ-treated diabetic rats.

**Conclusions:**

These findings suggest that insulin deficiency, rather than decreased physical activity, is a major contributor to early muscle atrophy in this model. Voluntary exercise alone is insufficient to prevent STZ-induced muscle atrophy in the absence of insulin. Leucine supplementation may offer a potential therapeutic strategy for muscle preservation.

## Highlights

• STZ-induced diabetes rapidly induces fast-twitch muscle atrophy.
• Insulin preserves muscle mass not solely through its glucose-lowering effects.
• Voluntary exercise alone is insufficient to prevent STZ-induced muscle atrophy in diabetic rats.
• STZ-induced diabetes alters skeletal muscle BCAA metabolism.
• Leucine supplementation attenuates muscle atrophy without affecting glucose level.

## Graphical abstract

**Figure:**
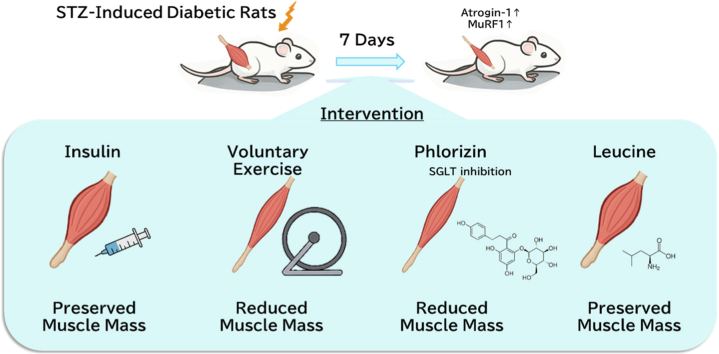

## 1 Introduction

Type 1 diabetes mellitus (T1DM) is characterized by autoimmune-mediated destruction of pancreatic β-cells, resulting in absolute insulin deficiency and chronic hyperglycemia. While insulin therapy is the cornerstone of T1DM management, the condition induces profound metabolic alterations beyond hyperglycemia, including accelerated skeletal muscle wasting. Muscle atrophy, particularly of fast-twitch fibers such as the extensor digitorum longus (EDL), contributes to reduced physical performance, increased frailty, and impaired glucose disposal, further exacerbating the diabetic state [1].

Insulin is not only a regulator of glucose metabolism but also a potent anabolic hormone essential for muscle maintenance. It promotes skeletal muscle protein synthesis through activation of the phosphoinositide 3-kinase (PI3K)/Akt/mTOR pathway and suppresses protein degradation by inhibiting muscle-specific E3 ubiquitin ligases such as Atrogin-1 and MuRF1 [2]. In insulin-deficient states, such as T1DM, the inhibition of these pathways results in a rapid shift toward catabolism and muscle mass loss. Notably, it remains unclear whether glycemic normalization alone is sufficient to preserve muscle mass or whether insulin signaling exerts additional effects beyond glucose lowering.

Exercise is a well-recognized intervention for improving insulin sensitivity and enhancing metabolic health in individuals with diabetes [1,3,4]. Physical activity stimulates skeletal muscle glucose uptake independently of insulin via AMP-activated protein kinase (AMPK)-dependent mechanisms and increases the expression and translocation of glucose transporter 4 (GLUT4) [5,6]. Chronic exercise also promotes mitochondrial biogenesis and muscle remodeling, supporting its potential utility in mitigating diabetic muscle loss [7]. However, it remains uncertain whether physical activity alone can counteract the catabolic effects of insulin deficiency in the absence of exogenous insulin therapy.

Previous studies have focused largely on structured exercise interventions. The potential of voluntary exercise to protect skeletal muscle mass during early stages of T1DM has not been fully explored. Moreover, the interaction between insulin replacement, glycemic control, and physical activity in preserving skeletal muscle remains poorly defined. To address these gaps, we investigated the impact of voluntary exercise on skeletal muscle atrophy in an STZ-induced T1DM rat model, utilizing a unique high-activity rodent strain (SPORTS rat) [[8], [9], [10], [11]]. We further assessed whether pharmacological glycemic control (SGLT inhibition) or nutritional intervention (leucine supplementation) could mitigate muscle loss, aiming to clarify the relative contributions of insulin, glucose control, and physical activity to muscle preservation during early diabetes.

## 2 Materials and methods

### 2.1 Animal studies

This study was performed in accordance with the guidelines for the care and use of laboratory animals of Tokushima University, and the protocol was approved by the Committee on Animal Research of Tokushima University Graduate School of Medicine. We used male Wistar rats (SLC, Inc. Shizuoka, Japan) and SPORTS rats at 6 weeks to 8 weeks of age, which were previously established in our laboratory [8]. Rats were housed at 22 °C to 24 °C and maintained on a 12-h-light, 12-h-dark cycle in the animal facility at Tokushima University Graduate School of Medicine. Male rats were injected intraperitoneally with sodium citrate (control) or 60 or 120 mg/kg STZ (Fujifilm Wako Pure Chemicals, Osaka, Japan), dissolved in 50 mM sodium citrate (pH 4.5) on day 0. Early-phase analyses were conducted on day 3 and day 7 after STZ injection, during which the wet weights of muscles, liver, and epididymal fat, as well as the expression of muscle atrophy-related genes, were evaluated. After 7 days, rats with a blood glucose level of greater than 300 mg/dL were considered diabetic, consistent with the early-phase analysis in this study, and this criterion was used to define animals for subsequent intervention studies. Blood glucose levels were measured after an overnight fast. To assess the role of insulin signaling in muscle preservation, one diabetic group received subcutaneous injections of intermediate-acting insulin (Novolin N, Novo Nordisk, Copenhagen, Denmark) at a dose of 12 units/day from Day 1 to Day 6 after STZ injection. To evaluate whether glycemic control alone could prevent muscle loss without insulin treatment, another diabetic group received the SGLT inhibitor phlorizin (Fujifilm Wako, Osaka, Japan) at a dose of 400 mg/kg/day, delivered subcutaneously twice daily from Day 1 to Day 6 after STZ injection. To explore the potential of nutrient-based therapy, a separate diabetic group was administered l-leucine orally (200 μL of 150 mM solution) once daily for 18 consecutive days, starting 7 days prior to STZ injection. SPORTS rats were housed in individual cages equipped with running wheels. Daily activity was continuously recorded for 7 days using a rotation-counting device. The overall experimental design and timeline are summarized in Supplemental Fig. 1.

### 2.2 RNA isolation and gene expression analysis

Total RNA was isolated from tissue using TRIzol reagent (Invitrogen, Carlsbad, CA, USA), and subjected to reverse transcription (RT) with a SuperScript VILO cDNA Synthesis kit (Invitrogen). The resulting cDNA was analyzed by quantitative polymerase chain reaction (qPCR) with SYBR Green PCR Master Mix in an ABI StepOne Plus Real-Time PCR system (Applied Biosystems). The abundance of target mRNAs was normalized to rat *Actb* mRNA as an internal control. The PCR primers (forward and reverse, respectively) were as follows: 5′-GAACAGCAAAACCAAAACTCAGTA-3′ and 5′- GCTCCTTAGTACTCCCTTTGTGAA-3′ for *Atrogin-1*, 5′-TGTCTGGAGGTCGTTTCCG-3′ and 5′-ATGCCGGTCCATGATCACTT-3′ for *Murf1*, 5′- CCCGCGAGTACAACCTTCT-3′ and 5′-CGTCATCCATGGCGAACT-3′ for *Actb*.

### 2.3 Oral glucose tolerance test

After an overnight fast, rats were orally administered a glucose solution (2 g/kg body weight) using a gavage needle. Blood was collected from the tail vein before administration (0 min) and 15, 30, 60, 90, and 120 min after administration. Blood glucose levels were measured using a glucose oxidase method (Glucocard, Arkray, Kyoto, Japan).

### 2.4 Metabolomics

Metabolomic profiling of skeletal muscle tissues was performed by capillary electrophoresis–time-of-flight mass spectrometry (CE-MS) with an Agilent 7100 CE system coupled to an Agilent 6230 TOF mass spectrometer (Agilent Technologies) according to previously described methods [12].

### 2.5 Statistical analysis

All data are expressed as mean ± SEM. Differences between groups were analyzed by the two-tailed unpaired Student's *t*-test, repeated-measures ANOVA, or one-way analysis of variance (ANOVA) with Bonferroni's post hoc test. Statistical significance was set at *P* < 0.05. Analyses were performed using GraphPad Prism 9.0 (GraphPad Software, CA, USA).

## 3 Results

### 3.1 STZ-induced diabetes caused rapid skeletal muscle atrophy

**Fig. 1:**
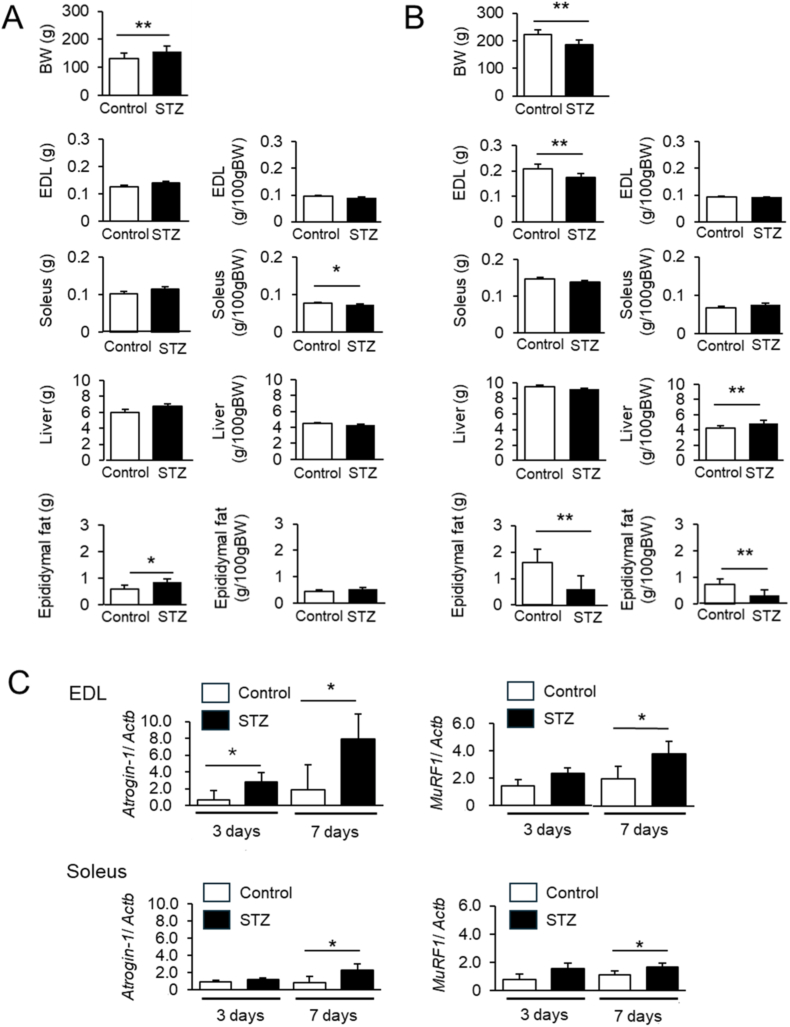
STZ-induced diabetes reduces skeletal muscle mass and upregulates atrophy genes. **(A)** Body weight (BW) and weights of EDL, soleus, liver, and epididymal fat in control and STZ-treated rats on day 3 post-injection. **(B)** BW and weights of EDL, soleus, liver, and epididymal fat in control and STZ-treated rats on day 7 post-injection. **(C)** Relative mRNA expression levels of Atrogin-1 and MuRF1 in EDL and soleus on days 3 and 7 post-STZ injection. Data are presented as mean ± SEM (*n*= 6 per group). ∗*P*< 0.05, ∗∗*P* < 0.01 by the two-tailed unpaired Student's *t*-test.

One week after STZ administration, diabetic rats exhibited significantly elevated blood glucose levels (>300 mg/dL) compared to controls. On day 3 post-STZ injection, body weight was not decreased, whereas the soleus muscle mass relative to body weight was reduced (Fig. 1A). On day 7 post-STZ injection, epididymal fat was reduced both in wet weight and relative to body weight, indicating systemic wasting (Fig. 1B). In contrast, although the wet weight of EDL was decreased, its relative weight was unchanged, likely reflecting concurrent body weight loss (Fig. 1B). Upregulation of muscle atrophy-related genes *Atrogin-1* and *Murf1* was observed as early as day 3 post-STZ administration, preceding measurable muscle mass loss (Fig. 1C). These data suggest that insulin deficiency triggers early activation of proteolytic pathways in glycolytic muscle.

### 3.2 Insulin, rather than SGLT inhibition, preserved muscle mass in diabetic rats

**Fig. 2:**
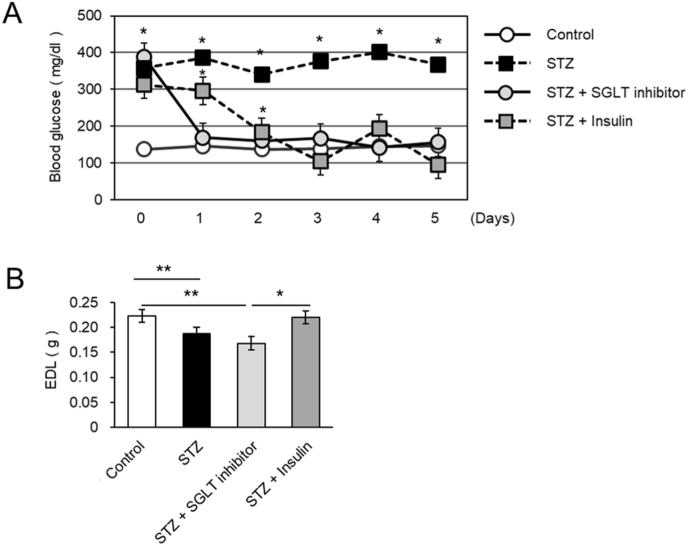
Insulin, but not an SGLT inhibitor, prevents EDL atrophy in diabetic rats. **(A)** Blood glucose levels in diabetic rats treated with insulin or an SGLT inhibitor over 7 days. **(B)** EDL muscle weights on day 7 in control rats (*n* = 6), STZ-treated rats (*n* = 6), STZ-treated rats with phlorizin (*n* = 5) and STZ-treated rats with insulin (*n*= 5). Data are presented as mean ± SEM. ∗*P* < 0.05 vs. control by repeated-measures ANOVA (A), or one-way ANOVA with Bonferroni's post hoc test (B).

To distinguish the role of insulin signaling from glucose lowering alone, diabetic rats were treated with either insulin or phlorizin. Blood glucose monitoring confirmed that both insulin and phlorizin effectively reduced hyperglycemia (Fig. 2A). Insulin treatment significantly attenuated EDL muscle loss compared to untreated diabetic rats (Fig. 2B). In contrast, despite improving blood glucose levels, phlorizin failed to prevent muscle atrophy. Similar results were observed when muscle weight was normalized to body weight (data not shown). These findings suggest that insulin signaling, rather than glycemic control alone, may contribute to the maintenance of muscle mass.

### 3.3 Voluntary physical activity was insufficient to prevent muscle atrophy in SPORTS rats

**Fig. 3:**
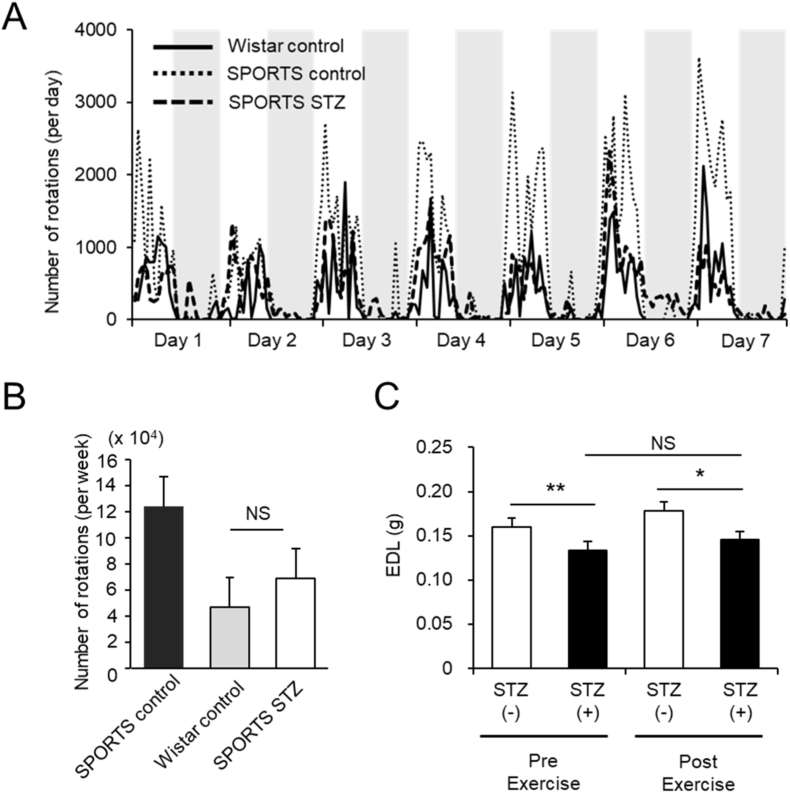
Voluntary wheel-running activity fails to prevent muscle atrophy in diabetic SPORTS rats. **(A)** Daily running activity in Wistar rats, SPORTS rats, and STZ-treated SPORTS rats over a 7-day period. **(B)** Cumulative weekly rotations in Wistar rats, SPORTS rats, and STZ-treated SPORTS rats. **(C)** EDL muscle weight on day 7 in SPORTS control rats (*n* = 9) and STZ-treated SPORTS rats (*n* = 11) without voluntary exercise, and in SPORTS control rats (*n* = 4) and STZ-treated SPORTS rats (*n*= 10) after voluntary exercise. Data are presented as mean ± SEM. ∗*P*< 0.05, ∗∗*P* < 0.01 and NS (not significant) by one-way ANOVA (B) or two-way ANOVA with Bonferroni's post hoc test (C).

To investigate whether physical activity could counteract diabetes-induced muscle loss, we used SPORTS rats, which exhibit high voluntary running behavior. STZ-treated SPORTS rats showed reduced daily running activity compared to non-diabetic SPORTS control rats. However, their activity levels remained relatively high and were comparable to those of non-diabetic Wistar control rats throughout the 7-day period (Fig. 3A and B). Despite preserved activity, STZ-treated SPORTS rats had significantly decreased EDL muscle mass compared to non-STZ-treated SPORTS rats (Fig. 3C). Similar results were observed when muscle weight was normalized to body weight (data not shown). These findings suggest that voluntary physical activity alone is insufficient to prevent muscle atrophy in the absence of insulin in this model.

### 3.4 STZ-induced diabetes altered BCAA metabolism in skeletal muscle, and leucine supplementation attenuated muscle loss

**Fig. 4:**
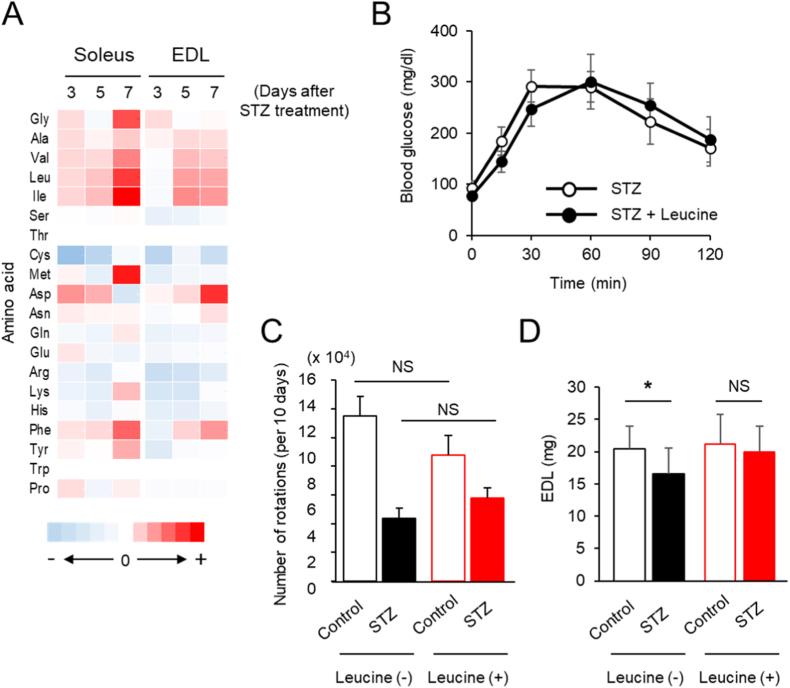
Leucine supplementation preserves EDL muscle mass in STZ-treated diabetic rats without altering blood glucose or physical activity. **(A)** Amino acid concentrations in EDL and soleus from STZ-treated diabetic rats assessed by CE-MS. **(B)** Oral glucose tolerance test after leucine supplementation. **(C)** Total 7-day wheel-running activity. **(D)** Effect of 18-day leucine supplementation on EDL muscle mass in control and STZ-treated rats. Data are presented as mean ± SEM (*n*= 4 per group). “+” and “-” indicate the direction of change in metabolite levels in STZ-treated rats relative to the control group, based on fold change values, with an absolute range of 0.39-fold to 1.88-fold in EDL and 0.10-fold to 2.09-fold in soleus (A). ∗*P* < 0.05 and NS (not significant) by two-way ANOVA (B), and two-way ANOVA with Bonferroni's post hoc test (C and D).

Metabolomic profiling of skeletal muscle revealed that STZ-induced diabetes significantly altered BCAA metabolism, particularly valine, leucine, and isoleucine, in both EDL and soleus muscle of diabetic rats compared to controls (Fig. 4A). These changes indicate disrupted amino acid metabolism during insulin-deficient diabetes and suggest potential metabolic compensatory mechanisms or impaired amino acid utilization. To test whether exogenous leucine could mitigate diabetes-induced muscle loss, diabetic rats were treated with oral leucine for 18 days. Leucine supplementation significantly preserved EDL muscle mass compared to diabetic controls, despite persistent hyperglycemia and no change in activity (Fig. 4B–D).

## 4 Discussion

This study suggests that insulin deficiency rapidly induces skeletal muscle atrophy in an STZ-induced model of T1DM, and that glycemic control through SGLT inhibition or high levels of voluntary physical activity alone may be insufficient to prevent muscle loss in this model. In contrast, insulin replacement significantly preserved skeletal muscle mass, particularly EDL muscle, indicating a critical role for insulin signaling in maintaining skeletal muscle integrity. Furthermore, we show that diabetes disrupts BCAA metabolism within skeletal muscle and that dietary leucine supplementation partially attenuates fast-twitch muscle atrophy, even in the absence of insulin.

The extreme activity levels of SPORTS rats may influence muscle weight, and their lean phenotype may reflect a qualitative metabolic adaptation rather than simple atrophy. Previous reports have indicated that, although SPORTS rats exhibit lower body weight and reduced abdominal fat, they maintain enhanced skeletal muscle oxidative capacity and energy homeostasis to sustain their hyperactive behavior [8,13]. In addition, previous studies have suggested potential alterations in skeletal muscle characteristics at the molecular level in SPORTS rats. Specifically, SPORTS rats appear to exhibit a fast-twitch–dominant profile compared with wild-type Wistar rats, with higher expression of Myh4 and Myh1 even under sedentary conditions. Furthermore, voluntary wheel running markedly upregulates these fast-twitch–related genes while downregulating slow-twitch fiber–related genes, suggesting a shift toward a fast-twitch phenotype with exercise [13]. These intrinsic characteristics may affect the susceptibility of fast-twitch muscles, such as EDL, to STZ-induced diabetes.

Insulin is well known for its anabolic role in skeletal muscle, promoting protein synthesis via the PI3K–Akt–mTOR pathway while simultaneously inhibiting proteolytic pathways regulated by E3 ubiquitin ligases such as Atrogin-1 and MuRF1 [2,14,15]. In our model, expressions of Atrogin-1 and MuRF1 were upregulated within three days of STZ administration, prior to measurable muscle loss. These findings suggest that muscle atrophy in early T1DM is driven primarily by suppressed insulin signaling rather than hyperglycemia alone. To explore this distinction, we compared insulin therapy with SGLT inhibition. While both interventions reduced blood glucose levels, only insulin treatment prevented EDL muscle loss. These data suggest that insulin's role in muscle preservation is not merely due to glycemic regulation but involves direct activation of muscle anabolic pathways.

Physical activity is widely regarded as a key component of diabetes therapy due to its insulin-independent effects on glucose uptake via AMPK and GLUT4 activation [5,6]. However, in this study, diabetic SPORTS rats—despite maintaining high levels of voluntary running—exhibited significant EDL muscle loss. These results suggest that exercise, in the absence of insulin, may be insufficient to counteract the strong catabolic signaling in skeletal muscle during early diabetes. This is consistent with the notion that voluntary physical activity may be insufficient to prevent STZ-induced muscle atrophy in the absence of insulin [16].

Our metabolomic analysis revealed elevated levels of BCAAs, including leucine, in diabetic skeletal muscle. Previous studies have suggested that accumulation of BCAAs in insulin-resistant or diabetic states may reflect impaired amino acid utilization or reduced oxidative capacity, rather than enhanced catabolism [17,18]. In addition, alterations in protein turnover, including increased proteolysis, may also contribute to elevated intracellular amino acid levels under catabolic conditions [17,18]. These findings may reflect reduced amino acid oxidation or altered protein turnover, as seen insulin-resistant states. Interestingly, exogenous leucine supplementation preserved EDL mass in diabetic rats. This suggests that leucine may play a protective role through mechanisms that are not fully dependent on insulin signaling; however, the underlying mechanisms remain unclear. Although leucine has been reported to stimulate muscle protein synthesis via mTOR signaling [19,20], the involvement of this pathway in the present findings remains speculative. These results highlight the potential for targeted nutritional strategies as adjunct therapies for diabetic sarcopenia, particularly when insulin therapy is limited or delayed.

Soleus muscle appeared relatively resistant to atrophy. A transient decrease was observed at an early stage prior to body weight loss, but this was not evident thereafter. This may be attributable to fiber type–specific characteristics, as the soleus is predominantly composed of oxidative (slow-twitch) fibers, which have higher mitochondrial capacity and greater resistance to metabolic stress and proteolysis [21]. Consistent with previous studies, oxidative muscles are generally less susceptible to atrophy under metabolic stress conditions [22,23].

This study has several limitations that should be considered. First, our analysis focused primarily on fast-twitch EDL muscle that is particularly sensitive to insulin deficiency [20]. Although soleus muscle mass was measured, detailed molecular and histological analyses of oxidative (slow-twitch) muscle fibers were not performed and may provide additional insights. In addition, our evaluation of muscle atrophy relied mainly on mRNA expression of Atrogin-1 and MuRF1, and we did not assess protein levels or functional markers of protein turnover, such as protein synthesis rates or autophagy-related pathways. These additional analyses would further clarify the mechanisms underlying muscle atrophy. Second, while the SPORTS rat model allowed us to evaluate the role of voluntary physical activity, this model does not replicate structured resistance or endurance training paradigms typically used in clinical or experimental exercise interventions. Moreover, potential strain-related differences between SPORTS rats and Wistar rats cannot be fully excluded, which may have influenced the observed outcomes. Baseline skeletal muscle characteristics of the SPORTS strain, including fiber type distribution, and myofiber cross-sectional area, were not evaluated in the present study and remain incompletely characterized. These factors may differ from those of standard Wistar rats and could influence the response to exercise. Third, although leucine supplementation showed protective effects on muscle mass, we did not directly assess downstream signaling pathways such as mTOR or protein synthesis rates, which would clarify the mechanistic basis of its benefit. Finally, the duration of our experimental window was limited to the early phase (7–18 days) following diabetes induction; whether these findings persist over longer periods or apply to established diabetes remains to be determined.

In conclusion, our findings demonstrate that insulin plays a key role in the prevention of skeletal muscle atrophy in early T1DM, and that glycemic control alone or voluntary physical activity may be insufficient to compensate for its absence. Leucine supplementation may offer a promising strategy to support muscle preservation under insulin-deficient conditions. Together, these findings highlight the potential need for integrated therapeutic approaches that combine insulin, nutrition, and physical strategies to prevent diabetic muscle wasting.

## Funding

This work was supported by grants from 10.13039/100016974The Food Science Institute Foundation (Ryoushoku-kenkyukai [K172400190]) and the 10.13039/100009950Ministry of Education, Culture, Sports, Science, and Technology of Japan to H.S. (Grant-in-Aid for Scientific Research B [23K24792]), and from the 10.13039/100009950Ministry of Education, Culture, Sports, Science, and Technology of Japan to K.N. (Grant-in-Aid for Scientific Research B [24K02859]). This work was carried out by the joint research program (Project No. 11023) of the Institute for Molecular and Cellular Regulation, Gunma University.

## CRediT authorship contribution statement

**Yuki Yamasaki:** Data curation, Formal analysis, Investigation, Methodology, Validation. **Kazuhiro Nomura:** Data curation, Formal analysis, Funding acquisition, Methodology, Validation, Writing – original draft. **Yuka Matsumoto:** Investigation. **Kana Fukuda-Takeji:** Investigation. **Yumiko Miyatake:** Investigation. **Naoya Suemasa:** Investigation. **Yuna Izumi-Mishima:** Investigation. **Sonoko Yasui-Yamada:** Investigation. **Masashi Kuroda:** Investigation. **Nagakatsu Harada:** Writing – review & editing. **Yasuo M. Tsutsumi:** Writing – review & editing. **Tadahiro Kitamura:** Writing – review & editing. **Rie Tsutsumi:** Writing – review & editing. **Yutaka Nakaya:** Supervision, Writing – review & editing. **Hiroshi Sakaue:** Conceptualization, Funding acquisition, Project administration, Supervision, Writing – review & editing.

## Declaration of competing interest

The authors declare that they have no known competing financial interests or personal relationships that could have appeared to influence the work reported in this paper.

## Data Availability

Data will be made available on request.

## References

[1] Smith J.A.B., Murach K.A., Dyar K.A., Zierath J.R. (2023). Exercise metabolism and adaptation in skeletal muscle. Nat. Rev. Mol. Cell Biol..

[2] Glass D.J. (2003). Signalling pathways that mediate skeletal muscle hypertrophy and atrophy. Nat. Cell Biol..

[3] Colberg S.R., Sigal R.J., Yardley J.E., Riddell M.C., Dunstan D.W., Dempsey P.C., Horton E.S., Castorino K., Tate D.F. (2016). Physical activity/Exercise and diabetes: a position statement of the American diabetes association. Diabetes Care.

[4] Hawley J.A., Hargreaves M., Joyner M.J., Zierath J.R. (2014). Integrative biology of exercise. Cell.

[5] Richter E.A., Hargreaves M. (2013). Exercise, GLUT4, and skeletal muscle glucose uptake. Physiol. Rev..

[6] Krook A., Wallberg-Henriksson H., Zierath J.R. (2004). Sending the signal: molecular mechanisms regulating glucose uptake. Med. Sci. Sports Exerc..

[7] Egan B., Zierath J.R. (2013). Exercise metabolism and the molecular regulation of skeletal muscle adaptation. Cell Metab..

[8] Morishima-Yamato M., Hisaoka F., Shinomiya S., Harada N., Matoba H., Takahashi A., Nakaya Y. (2005). Cloning and establishment of a line of rats for high levels of voluntary wheel running. Life Sci..

[9] Morishima M., Harada N., Hara S., Sano A., Seno H., Takahashi A., Morita Y., Nakaya Y. (2006). Monoamine oxidase A activity and norepinephrine level in hippocampus determine hyperwheel running in SPORTS rats. Neuropsychopharmacology.

[10] Miyatake Y., Shiuchi T., Mawatari K., Toda S., Taniguchi Y., Futami A., Sato F., Kuroda M., Sebe M., Tsutsumi R., Harada N., Minokoshi Y., Kitamura T., Gotoh K., Ueno M., Nakaya Y., Sakaue H. (2017). Intracerebroventricular injection of ghrelin decreases wheel running activity in rats. Peptides.

[11] Nomura K., Yamasaki Y., Takeji K., Deha S., Yamashita K., Izumi-Mishima Y., Yasui-Yamada S., Kuroda M., Harada N., Kitamura T., Tsutsumi Y.M., Tsutsumi R., Nakaya Y., Sakaue H. (2025). Gut-pancreas axis in the control of insulin secretion in streptozotocin-resistant rats. Biochem. Biophys. Res. Commun..

[12] Okamatsu-Ogura Y., Kuroda M., Tsutsumi R., Tsubota A., Saito M., Kimura K., Sakaue H. (2020). UCP1-dependent and UCP1-independent metabolic changes induced by acute cold exposure in brown adipose tissue of mice. Metabolism.

[13] Horiguchi T., Miyatake Y., Miyoshi K., Tanimura A., Hagita H., Sakaue H., Noma T. (2020). Gene-expression profile reveals the genetic and acquired phenotypes of hyperactive mutant SPORTS rat. J. Med. Investig..

[14] Sandri M., Sandri C., Gilbert A., Skurk C., Calabria E., Picard A., Walsh K., Schiaffino S., Lecker S.H., Goldberg A.L. (2004). Foxo transcription factors induce the atrophy-related ubiquitin ligase Atrogin-1 and cause skeletal muscle atrophy. Cell.

[15] Bodine S.C., Latres E., Baumhueter S., Lai V.K.-M., Nunez L., Clarke B.A., Poueymirou W.T., Panaro F.J., Na E., Dharmarajan K., Pan Z.-Q., Valenzuela D.M., DeChiara T.M., Stitt T.N., Yancopoulos G.D., Glass D.J. (2001). Identification of ubiquitin ligases required for skeletal muscle atrophy. Science.

[16] Sylow L., Tokarz V.L., Richter E.A., Klip A. (2021). The many actions of insulin in skeletal muscle, the paramount tissue determining glycemia. Cell Metab..

[17] Newgard C.B. (2012). Interplay between lipids and branched-chain amino acids in development of insulin resistance. Cell Metab..

[18] Vanweert F., Schrauwen P., Phielix E. (2022). Role of branched-chain amino acid metabolism in the pathogenesis of obesity and type 2 diabetes-related metabolic disturbances BCAA metabolism in type 2 diabetes. Nutr. Diabetes.

[19] Greiwe J.S., Kwon G., McDaniel M.L., Semenkovich C.F. (2001). Leucine and insulin activate p70 S6 kinase through different pathways in human skeletal muscle. Am. J. Physiol. Endocrinol. Metabol..

[20] Liu Z., Wu Y., Nicklas E.W., Jahn L.A., Price W.J., Barrett E.J. (2004). Unlike insulin, amino acids stimulate p70^S6K^ but not GSK-3 or glycogen synthase in human skeletal muscle. Am. J. Physiol. Endocrinol. Metabol..

[21] Schiaffino S., Reggiani C. (2011). Fiber types in mammalian skeletal muscles. Physiol. Rev..

[22] Sandri M. (2008). Signaling in muscle atrophy and hypertrophy. Physiology.

[23] Wang X.H., Mitch W.E. (2014). Mechanisms of muscle wasting in chronic kidney disease. Nat. Rev. Nephrol..

