# Supplementary figures and images for: Insulin preserves fast-twitch muscle mass during early STZ-induced diabetes in rats
Source: Biochem Biophys Rep. 2026 May 8;46:102627. doi: 10.1016/j.bbrep.2026.102627 (PMC13186084; doi:10.1016/j.bbrep.2026.102627)

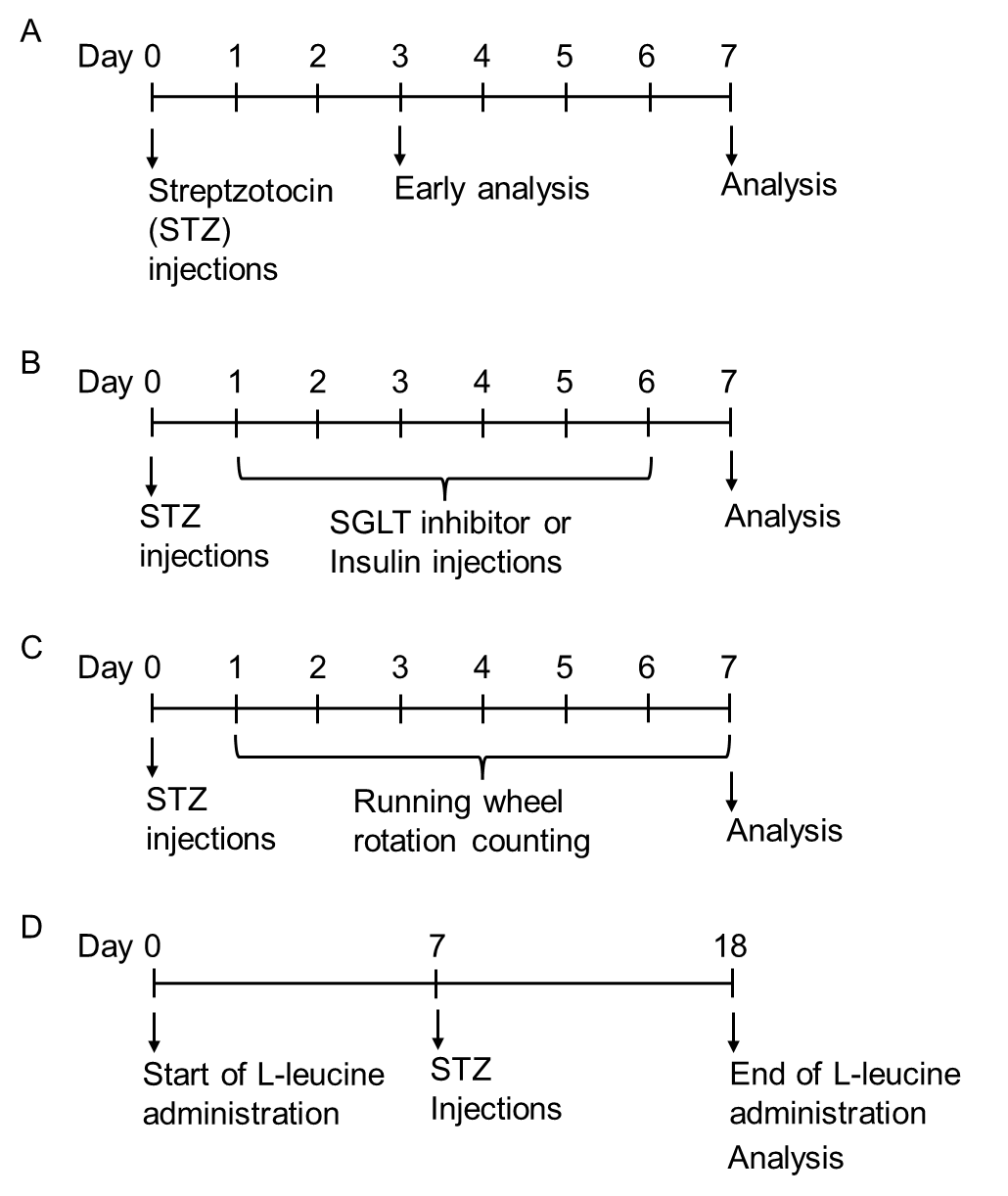
**Supplemental Figure 1**

Supplement: Multimedia component 1 [file mmc1.docx]
